# Dr. Addison on Neuralgic Pains

**Published:** 1830-07-01

**Authors:** 


### Uterine Source of Neuralgic Pains.*

The following case, taken from the books of Guy's Hospital, is adduced by Dr. Addison, as an illustration of the connexion between the condition of the uterus and local neuralgic pains.

"Elizabeth Harris, ætat. twenty-six, a woman of irritable habit, subject to frequent pain of the head and left side, with palpitations of the heart on the least exertion—her menstrual periods are regular, and generally attended with but little pain, but the discharge is usually rather profuse, with passage of clots, and lasting a week. Last Tuesday, while the catamenia were flowing, accidentally got wet by upsetting a tub of ice-water over her legs and feet; the discharge ceased almost immediately, but she did not feel any inconvenience until Wednesday afternoon, when she was seized with pain, first commencing at the scrobiculus cordis, but soon became fixed at the lower part of the abdomen. This pain gradually becoming more acute, she came into the hospital on Thursday afternoon (February 11th.) At that time it was very severe; the abdomen acutely tender to the touch on first making pres-

* Dr. Addison.

sure upon it, but after continuing the pressure it was tolerated. Bowels had not been relieved; pulse 120, but without jerk; tongue slightly furred.'

"When I first saw her she could not bear even slight pressure on any part of the belly, and as she was rather of a full habit of body, I ordered her to lose sixteen ounces of blood—to take a smart dose of Colocynth and Calomel, and to have the belly fomented with the Chamomile fomentation. As I was satisfied as to the character of the pain, you might perhaps have expected me to order, with moderate depletion, some form of opiate, either by the mouth or by glyster, with a view to relieve the violence of the pain; whilst you were probably surprised at my giving the Colocynth and Calomel, as I have elsewhere said that such a combination usually irritates, and causes an increase of pain in such subjects. I adopted the practice, however, with a hope that this aloetic compound might, in conjunction with the bleeding and fomentation, bring about a return of the menstrual discharge, which I calculated, with tolerable certainty, would afford speedy relief. In the evening she experienced great aggravation of her pain, evidently from the irritation of the pills, so that at nine o'clock Mr. Dashwood gave her six drams of Castor Oil, applied thirty leeches to the belly, and repeated the fomentation. On the following day, her bowels having been freely opened, she obtained considerable relief, the severe pain only coming on at intervals, and being chiefly felt in the region of the sigmoid flexure. On the evening of the 13th, or the third day from her admission, *she had a slight return of the catamenia, and next morning we found her entirely free from pain.* In the evening, however, *they again ceased,* and at two o'clock in the morning *the pain again returned* in the lower belly. This pain gradually subsided under the treatment adopted, but on the 17th she was represented to be suffering from *acute pain under the margin of the ribs of the right side.* This was supposed by some to be pleuritic, but although it was manifestly increased by inspiration, it varied very much in its intensity, and at intervals was very much aggravated, which, with the other symptoms and history of the case, left no doubt in my mind that it was abdominal and purely neuralgic. I applied a blister and gave the Conium. On the following morning she was nearly altogether free from pain."

In the above case, Dr. A. thinks it is impossible not to conclude that the state of the uterus and the neuralgic pain in the abdomen are to be viewed in the light of cause and effect, however that causation may be brought about. The case, indeed, is familiar to the observation of those who have opportunities of seeing the consequences of sudden suppression of the lochia shortly after delivery—the check being frequently followed by a severe neuralgic pain attacking the abdomen, more or less extensively in different individuals. Dr. A. does not think it improbable that some of those cases which have been published as anomalous forms of puerperal fever, may have partaken of the same nature as the painful affections of the abdomen to which he has directed the attention of his pupils and the public in the work which he has recently published, and of which we have given an analysis in another part of the Journal.

---

